# A Chemiluminescent Magnetic Enzyme Immunoassay Method for 2 Triazole Pesticide Detection in Wheat

**DOI:** 10.3390/foods15030577

**Published:** 2026-02-05

**Authors:** Xin Shi, Kai Huang, Baoyuan Guo, Xinbao Liu, Hongmei Liu, Wei Zhang, Yang Wang, Zhe Wang, Chun’e Zhang

**Affiliations:** 1 School of Health Science and Engineering, University of Shanghai for Science and Technology, Shanghai 200093, China; shixin7126@163.com (X.S.); hkjn1990@163.com (K.H.); gby@ags.ac.cn (B.G.); 2 Academy of National Food and Strategic Reserves Administration, Beijing 100037, China; lhm@ags.ac.cn (H.L.); zw@ags.ac.cn (W.Z.); wy@ags.ac.cn (Y.W.); 3 Ningxia Hui Autonomous Region Grain and Oil Product Quality Inspection Center, Yinchuan 750001, China; 15209610781@163.com

**Keywords:** wheat, triazolone, tebuconazole, chemiluminescence, magnetic enzyme immunoassay

## Abstract

We developed an alkaline phosphatase (AP) chemiluminescence immunoassay method by combining the superparamagnetic magnetic beads and the biotin–streptavidin signal amplification system to detect the triazolone and tebuconazole in wheat. Through optimization of the extraction solution and extraction time, acetonitrile–PBS was selected as the extraction solution with an extraction time of 5 min as the optimal pretreatment condition. Optimizing the dilution ratio of antigen antibodies, the optimal detection conditions were selected as the dilution ratios of 1:8000 and 1:20,000 for the triazolone monoclonal antibody solution and biotinylated triazolone solution, and 1:4000 and 1:20,000 for the tebuconazole monoclonal antibody solution and biotinylated tebuconazole solution, respectively. Under the optimal conditions, the method demonstrated that the limits of detection (LOD) of triazolone and tebuconazole were 0.002835 μg·mL^−1^ and 0.00064 μg·mL^−1^, respectively. The recovery rate was between 90.1% and 103.6%, and the relative standard deviation (RSD) was lower than 10%. The cross-reaction rates for structural analogs were all less than 0.1%, showing good specificity. In actual sample detection, this method did not detect triazolone and tebuconazole, and the results were consistent with UHPLC-MS/MS.

## 1. Introduction

**Figure 1:**
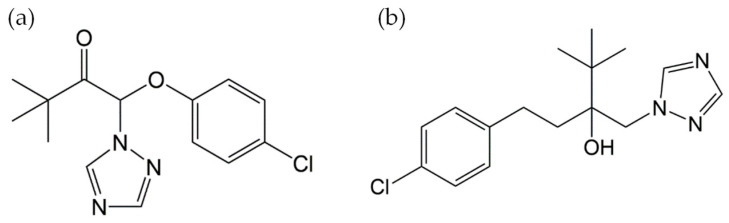
Structural formula of triazolone and tebuconazole. (**a**) Triazolone and (**b**) tebuconazole.

Triazole fungicides are a broad-spectrum class of agricultural chemicals widely used to control fungal diseases in crops. Given the potential health risks to humans and animals, the residues of triazoles in foodstuffs are a key focus in regulation [1,2,3]. As a staple grain, wheat is often threatened by fungal pathogens that cause powdery mildew, rust, black rot, and leaf blight [4]. Triazole fungicides, such as triazolone and tebuconazole (Figure 1), are widely used to combat these phytopathies [5,6,7]. These compounds are rapidly absorbed by the plant tissues and inhibit the biosynthesis of ergosterol in target fungi, providing both protective and curative effects [8,9,10]. However, the improper or excessive application might lead to elevated residue levels and disseminate into soil, water, and air, posing potential risks to human health and ecosystems [11,12,13,14].

The regulators have established the maximum residue limit (MRL) for triazole fungicides in grain to ensure food safety and guide the analytical method development [15]. EU regulations 2016/103 and 2017/627 set the MRLs for tebuconazole and triazolone in wheat at 0.05 mg/kg, while GB 2763–2021 standard [16] specifies limits of 0.05 mg/kg for tebuconazole and 0.2 mg/kg for triazolone in China, respectively. To enforce these standards, reliable detection methods are essential for verifying compliance. Conventional analysis relies on liquid or gas chromatography techniques [17,18,19]. For instance, ultra-high-performance liquid chromatography–tandem mass spectrometry [20] and gas chromatography–ion trap mass spectrometry [21] have been employed for triazole detection in grains accurately and effectively. However, these methods require long analysis time and high operating costs, which limit their practical utility [22,23,24,25,26].

Chemiluminescence enzyme immunoassay (CLEIA) utilizes antigen–antibody recognition for quantitative detection, offering high sensitivity, rapid analysis, broad linear range, and cost-effectiveness for pesticide residue monitoring [27,28,29,30]. It typically employs enzyme-labeled antibodies to transduce immune binding into amplified chemiluminescent signals. While the biotin–streptavidin system (BSS) could further enhance sensitivity via high-affinity binding [31,32], traditional manual protocols involving centrifugation and repeated washing steps are time-consuming. Moreover, these manual operations suffer from poor reproducibility, primarily due to analyte loss and residual contamination during the washing process [33].

Magnetic microsphere-based CLEIA addresses these limitations by employing superparamagnetic beads as solid-phase carriers. The beads capture target complexes, preventing dispersion while enabling precise magnetic manipulation [34]. Su et al. [35] developed a novel sandwich-type magnetic polymer microsphere modified with dextrose and demonstrated that magnetic PFS-PGDC microspheres can significantly reduce the adsorption rate of non-specific proteins by over 90%, demonstrating significant advantages in improving the sensitivity of chemiluminescence analysis. Zhao et al. [36] used poly microspheres as a novel signal enhancer, and the sensitivity far exceeded that achieved by classical chemiluminescence immunoassay. Critically, the superparamagnetic properties allow for achieving automation by using an automated chemiluminescence immunoassay analyzer. The analyzer-controlled electromagnet arrays execute bead capture, washing, and transfer without manual interference, eliminating human intervention [37,38].

In this study, a novel chemiluminescence immunoassay method was developed for analyzing triazole pesticides in wheat. Magnetic microspheres served as the core solid-phase carrier, enabling automated high-throughput separation and target capture while leveraging the advantages of processing. By integrating the biotin–streptavidin system with alkaline phosphatase-assisted signal amplification, the sensitivity of the indirect competitive immunoassay was enhanced. The extraction conditions and antigen/antibody dilution ratios were optimized to reduce the interference of the complex matrix and improve the sensitivity. Six samples were automatically processed within 30 min, eliminating manual errors and improving efficiency, accuracy, and reproducibility by using a chemiluminescence immunoanalyzer. Through reliable system evaluation, this method provides technical support for pesticide residue monitoring and quality control in agricultural products.

## 2. Materials and Methods

### 2.1. Materials and Reagents

Triazolone and tebuconazole standard products were purchased from Tianjin Alta Technology Co., Ltd. (Tianjin, China). Diclofenac, tebuconazole, and pentachloroaniline were purchased from Shanghai Yuanye Biotechnology Co., Ltd. (Shanghai, China). Dichlorvonitrile and Chlorpromazine were purchased from Tianjin Alta Technology Co., Ltd. (Tianjin, China). Alkaline phosphatase-labeled goat anti-mouse IgG (H+L) was purchased from Shanghai Biyuntian Biotechnology Co., Ltd. (Shanghai, China). Streptavidin and carboxyl magnetic beads were purchased from Suzhou Beaver Biomedical Engineering Co., Ltd. (Suzhou, China). Antigens were purchased from Beijing Xinshengke Technology Co., Ltd. (Beijing, China). Biotin was purchased from Nanjing Jiaoziteng Scientific Equipment Co., Ltd. (Nanjing, China). Monoclonal antibody was purchased from Shandong Landu Biotechnology Co., Ltd. (Binzhou, China). Alkaline phosphatase luminescent substrate 01 was purchased from Beijing Aiweide Biotechnology Co., Ltd. (Beijing, China). Phosphate-buffered solution (PBS), acetonitrile (CH_3_CN), and methanol (CH_3_OH) were purchased from Beijing Deyi Hengda Biotechnology Co., Ltd. (Beijing, China). Tween 20, Proclin 300, phosphate-buffered saline (PBS), and tris-buffered saline (TBS) salt packs were provided by Beijing Biotopped Technology Co., Ltd. (Beijing, China). All other chemical reagents were obtained from Sangon Biotechnology Co., Ltd. (Shanghai, China).

Wheat blank samples were purchased from the Academy of National Food and Strategic Reserves Administration (Beijing, China). Five wheat samples were collected from Shandong Province and Henan Province, respectively. Ten wheat samples were collected in the field.

### 2.2. Instruments and Equipment

The Mettler Toledo electronic analytical balance (Mettler Toledo Instruments (Shanghai, China) Co., Ltd.) was used to weigh samples. The multi-tube vortex mixer (Hangzhou Ausheng Instrument Co., Ltd., Hangzhou, China) was used to extract triazolone and tebuconazole from the sample. The magnetic separator (Kangyuan Biotechnology, Beijing, China) was used to collect carboxyl magnetic beads. The high-speed centrifuge (Xiangyi Centrifuge Instrument Co., Ltd., Changsha, China) was used for the purification of biotinylated antigens. The SCIEX 6500+ QTRAP™ UHPLC-MS/MS system (AB Sciex Co., Ltd., Framingham, MA, USA) equipped with an electrospray ionization (ESI) source was used for UHPLC-MS/MS analysis. The FEI Talos F200X transmission electron microscope (Thermo Fisher Scientific Co., Ltd., Middlesex, MA, USA) and the ZEISS Sigma 360 scanning electron microscope (Carl Zeiss AG Co., Ltd., Oberkochen, Germany) were used to detect the morphological characteristics of modified magnetic microspheres. The chemiluminescence immunoassay analyzer (Beijing Meilian Taike Biotechnology Co., Ltd., Beijing, China.) was used to test triazolone and tebuconazole in the sample.

### 2.3. Synthesis and Characterization of Magnetic Bead-Labeled Streptavidin (MB-SA)

The carboxyl magnetic beads (1 mL) were washed three times with 0.1 M MES (pH 4.5). After magnetic separation, the beads were activated by adding 0.5 mL EDC (0.5 mg/mL) and 0.5 mL NHS (0.5 mg/mL). After shaking for 30 min, the supernatant was discarded, and the activated beads were incubated with 1 mg streptavidin in 1 mL PBS for 2 h. After magnetic separation, the beads were blocked with 1.0 mL Tris buffer for 60 min, then washed three times with 2 mL phosphate-buffered solution (0.05 mol/L, pH 7.4) containing 0.1% Tween 20 (*v*/*w*) (PBST). Finally, the streptavidin-modified beads were resuspended in 1 mL phosphate-buffered solution (0.05 mol/L, pH 7.4) containing 0.02% sodium azide and stored at 4 °C for use.

### 2.4. Preparation of Biotinylated Antigens

The antigen–BSA was dissolved in PBS to a final concentration of 2 mg/mL and reacted with the NHS–PEG_4_–biotin solution of 10 mM for 30 min at room temperature. The biotin-labeled conjugate was purified using an ultrafiltration tube at 8000 rpm three times.

### 2.5. Sample Pretreatment and Optimization

Five grams (±0.05 g) of each finely ground wheat sample was vortex-extracted with 20 mL of extraction solution at 2500 rpm. The supernatant was collected and filtered through a 0.8 μm filter membrane for analysis. Since the extraction solution and extraction time affect both efficiency and detection sensitivity, the extraction solutions of methanol-PBS (20:80, *v*/*v*), acetonitrile–PBS (20:80, *v*/*v*), and 0.01 M PBS, for the extraction times of 5, 10, and 25 min, were used for analysis.

### 2.6. Optimization of Reaction Conditions

The performance of MB-based CLIA is related to various preparation conditions, and optimizing the relevant parameters of the reaction system can improve the detection of pesticide residue content. The chessboard method was used to optimize the dilution ratio of the antibody solution and biotinylated antigen solution. Both solutions were diluted to four concentrations, respectively [39]. The triazolone monoclonal antibody solution was subjected to serial dilutions at ratios of 1:2000, 1:4000, 1:6000, and 1:8000. Moreover, the biotinylated triazolone solution was subjected to serial dilutions at ratios of 1:10,000, 1:12,500, 1:15,000, and 1:20,000. The tebuconazole monoclonal antibody solution was subjected to serial dilutions at ratios of 1:1000, 1:2000, 1:4000, and 1:6000. In addition, the biotinylated tebuconazole solution was subjected to serial dilutions at ratios of 1:5000, 1:10,000, 1:15,000, and 1:20,000. The signal value B of both pesticide quality control samples and the signal value B_0_ of the blank samples were measured, respectively, and the ratio of B/B_0_ was calculated.

### 2.7. The MB-Based CLIA Detection

**Figure 2:**
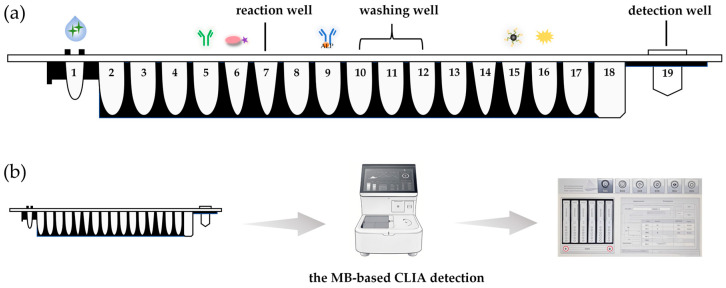
CLIA detection method based on MB. (**a**) Detection process of the proposed method and (**b**) schematic diagram of the reagent strip.

As shown in Figure 2a, first, the following liquids were preloaded into the 7 wells of the reagent strip: 70 μL of monoclonal antibody solution, 70 μL of biotinylated antigen solution, 70 μL of alkaline phosphatase-labeled IgG solution (AP-IgG), 350 μL of TBST buffer, 130 μL of Streptavidin-labeled magnetic bead (MB-SA) solution, and 200 μL of chemiluminescent substrate APS-5 solution. After that, 50 μL of the test sample solution was added to the sample well to complete the construction of the reaction system. The transfer of solution was completed based on the linear module of a three-axis robotic arm. The magnetic rod and stirring sleeve were manipulated by the MB control module to realize manipulation of magnetic beads, such as magnetic separation, transfer, and resuspension. The process of the MB-based CLIA consisted of the following steps, as shown in Figure 2b:

1. Through the automatic transfer module, 150 μL of APS-5 substrate (in well 16) was transferred to the detection well (in well 19); 20 μL of sample (in well 1), c (in well 5), and 50 μL of AP-IgG (in well 9) were sequentially transferred to the reaction well (in well 7). After mixing the magnetic beads in well 15, 100 μL was transferred to the reaction well, and finally, 50 μL of biotinylated antigen solution in well 6 was transferred to the reaction well and incubated for 7 min.
2. In the reaction well, a stirring sleeve was placed into the solution and moved up and down at room temperature for 5 min (stirring). Subsequently, by moving the magnetic rod into the stirring sleeve and collecting for 1 min (magnetic separation), the MB-SA–biotin–antigen–mAb-IgG-AP complex at the bottom of the stirring sleeve was collected. Finally, the magnetic rod and stirring sleeve were retracted, and the composite was transferred to the washing hole (in well 10) by parallel movement (transfer) of the magnetic rod. In the washing well, the magnetic rod and stirring sleeve were placed into the solution, and then the magnetic beads were released by removing (suspending) the magnetic rod from the stirring sleeve. The stirring sleeve moved up and down rapidly (100 times per minute) for 2 min (washing), followed by magnetic separation and transfer to the next washing well (in well 11). The complex in the reaction well was washed three times (in wells 10, 11, and 12) with the assistance of a built-in magnet in the instrument to remove excess reagents and non-specific binding, and then transferred to the detection well for the AP-catalyzed chemiluminescence reaction.
3. The luminescence value was evaluated. The analytical results were shown on the screen.

### 2.8. Validation of the MB-Based CLIA

#### 2.8.1. Establishment of Working Curve

The extraction solution was added to the blank wheat matrix samples, followed by vortex extraction. After settlement, the supernatant was collected and filtered using a 0.8 μm microporous membrane. The triazolone and tebuconazole were added to the supernatant to prepare a series of standard solutions with concentration gradients, respectively. RLU was measured, and the standard curve was constructedusing a nonlinear four-parameter fitting function.

#### 2.8.2. Sensitivity Evaluation

Twenty blank wheat samples were pre-treated and analyzed by chemiluminescence immunoassay. The LOD was calculated by the formula LOD = X + 3 × SD, and the LOQ was calculated by LOQ = X + 10 × SD, respectively, where X is the mean value of 20 blank wheat samples, and SD is the standard deviation of 20 test samples.

#### 2.8.3. Precision Evaluation

The triazolone and tebuconazole standard solutions at high, medium, and low levels were added to the supernatant of blank wheat samples. The chemiluminescence signal of the solution with different pesticide concentrations was measured. The average RLU and the relative standard deviation RSD were calculated to evaluate the precision of the method.

#### 2.8.4. Accuracy Evaluation

The supernatant of blank wheat samples was spiked at three levels: half the international maximum residue limit, at the maximum residue limit, and two times the international maximum residue limit. The samples were analyzed by both UHPLC-MS/MS and chemiluminescence methods, and the accuracy of the methods was evaluated by calculating the average recovery rate and CVs. As shown in Table 1, UHPLC-MS/MS analysis was performed on a SCIEX 6500+ QTRAP™ MS/MS system operating in multiple reaction monitoring (MRM) mode and in positive mode with following source parameters: curtain gas 40, ion spray voltage 5500 V, source temperature 500 °C, gas 1 and 2 both at 40 au, and CAD gas set to medium.

Triazolone and tebuconazole were separated on a Kinetex C18 column (100 × 2.1 mm, 1.8 μm, Phenomenex). The elutes were (A) water (0.1% FA, 1 mM NH_4_FA) and (B) methanol. The elution program was started with 10% B, after kept for 1 min, the proportion of B was linearly increased to 90% in 9.0 min, which was held for 3 min. The proportion of B was then decreased back to 10% in 0.5 min and kept for 3 min to equilibrate the column. The flow rate was set to 0.3 mL/min, and the column temperature was 40 °C. The injection volume was 2 μL.

#### 2.8.5. Selectivity Evaluation

Chlorthal, dichlorvonitrile, difenoconazole, diniconazole, pentachloroaniline, and other analogs were selected as competitors and tested as described in Section 2.7. The IC_50_ value was calculated for each compoundand the cross reactivity rate (CR) was calculated as Equation (1):

(1) $$CR=\frac{{IC}_{50\;target}}{{IC}_{50\;other}}\times 100\%$$

where IC_50_ target represents 50% inhibition concentration of pesticides. IC_50_ other represents 50% of the inhibitory concentration of other analogs.

#### 2.8.6. Wheat Sample Detection

Based on the optimized pre-processing conditions and detection methods mentioned above, the ten purchased samples were tested and validated against the results of the UHPLC-MS/MS detection method. According to GB 23200.121–2021 [40], the content of triazolone and tebuconazole in wheat is determined by UHPLC-MS/MS. The sample (5 ± 0.01 g) was weighed in a 50 mL centrifuge tube, mixed with 10 mL of water vortex, and allowed to stand for 30 min. Acetonitrile–acetic acid solution (15 mL) and a ceramic proton were added and shaken for 1 min. Anhydrous magnesium sulfate (6 g) and sodium acetate (1.5 g) were added and shaken for 1 min, then centrifuged for 5 min (4200 r/min). The supernatant was quantitatively aspirated into a centrifuge tube containing a water removal agent and purification material, vortex mixed for 1 min, and centrifuged for 5 min (4200 r/min). The supernatant was extracted and filtered through a microporous membrane for testing.

## 3. Results

### 3.1. Detection Principle

**Figure 3:**
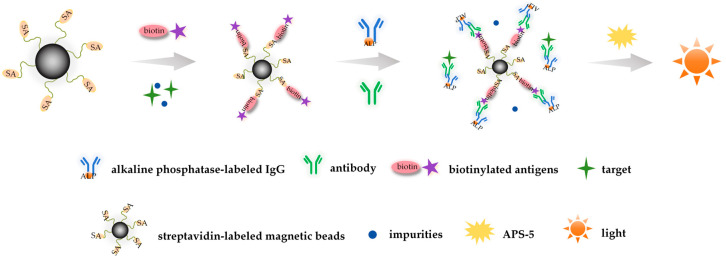
Schematic diagram of detection principle.

The detection system employed the competitive immunoassay, as shown in Figure 3. Specific antibodies bind to alkaline phosphatase (AP)-labeled immunoglobulin, forming immune complexes that compete with target analytes for binding to the biotinylated antigens. Streptavidin-modified magnetic beads (MB-SAs) serve as solid-phase carriers to capture these complexes, enabling specific signal amplification through the MB-SA–biotin interaction. Following magnetic separation and washing steps, AP catalyzed the chemiluminescence substrate (APS-5) to generate a photon signal; the emitted photons were quantified in relative light units (RLUs), and the detection signal was negatively correlated with the concentration of the target substance [41].

### 3.2. Characterization of Magnetic Beads

**Figure 4:**
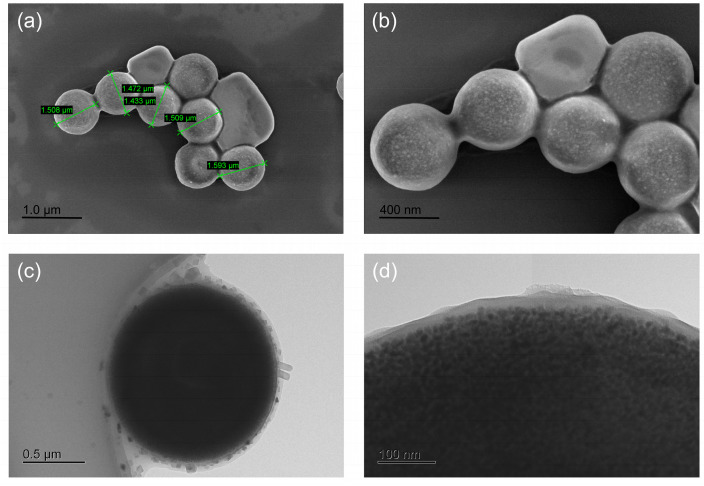
Characterization of MB-SA. (**a**,**b**) SEM image of the prepared MB-SA. (**c**,**d**) TEM image of the prepared MB-SA.

The morphology and particle size distribution of MB-SA were evaluated by SEM. As shown in Figure 4a,b, MB-SA is a uniformly sized spherical shape with a particle size of approximately 1.5 μm. The uniform particle size and regular morphology of MB-SA could ensure good repeatability in detection. The loading thickness of streptavidin on the MB-SA surface was evaluated by TEM. As shown in Figure 4c,d, streptavidin was loaded onto the surface of MB-SA with a thickness of approximately 20–30 nm. SEM and TEM images demonstrate the uniform morphology and complete surface coverage of the streptavidin layer, indicating stable attachment to the bead surface.

### 3.3. Optimization of Pre-Processing Conditions

#### 3.3.1. Optimization of Extraction Solvent

**Figure 5:**
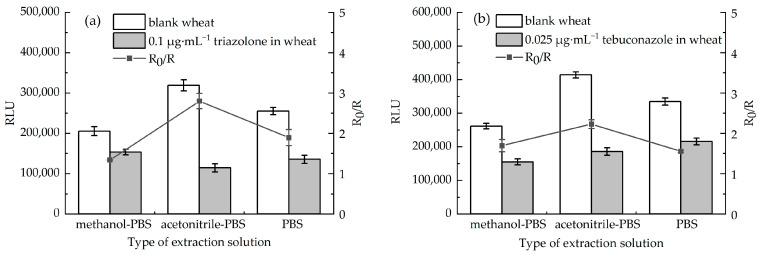
Effects of three extracts on RLU of two pesticides. (**a**) Triazolone and (**b**) tebuconazole.

There are mycotoxins, heavy metals, endogenous macromolecules, or other matrix interferences in the wheat extract that could interfere with the antigen–antibody binding and thus affect chemiluminescence immunoassay performance. To mitigate these interferences, the extraction solvent was optimized. Three different extraction solvent systems, including methanol–PBS (20:80, *v*/*v*), acetonitrile–PBS (20:80, *v*/*v*), and 0.01 M PBS, were evaluated. As shown in Figure 5, the acetonitrile–PBS (20:80, *v*/*v*) system has a significantly better extraction efficiency for the target triazole pesticides than the other two systems. It is speculated that its advantage stems from the precipitation effect of acetonitrile on interfering macromolecules in the wheat substrate, which can effectively reduce the co-extraction of substrate impurities. Meanwhile, the reasonable ratio with PBS can maintain the mild environment required for subsequent immune reactions, avoiding the damage to antibody activity caused by overly strong solvent polarity. Thus, while improving the extraction efficiency, it can also reduce the impact of substrate interference on chemiluminescence signals. Based on this, acetonitrile–PBS (20:80, *v*/*v*) was determined as the optimal extraction solvent for the subsequent analysis.

#### 3.3.2. Optimization of Extraction Time

**Figure 6:**
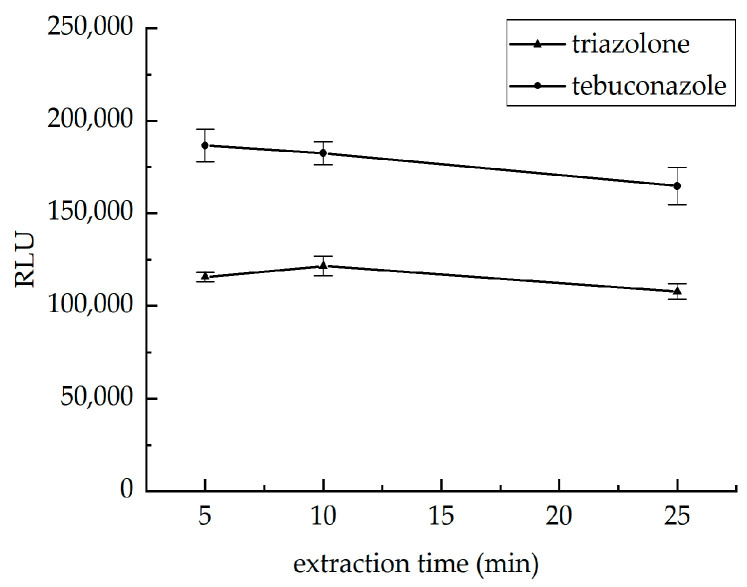
Effects of different extraction times on RLU of two pesticides.

To evaluate the effect of extraction time on the chemiluminescence signals, vortex extraction was performed for 5, 10, and 25 min. As shown in Figure 6, there was no significant difference in the chemiluminescence signal values measured at different extraction times, indicating that 5 min vortex extraction is sufficient to completely extract the target triazole pesticide. The reason is that the binding force between triazole pesticides and wheat substrate is weak, resulting in low desorption activation energy. The extraction solvent can quickly penetrate the substrate and achieve solid–liquid mass transfer equilibrium of the target substance within 5 min. Extending the extraction time does not significantly improve the recovery rate, but instead increases the co-extraction rate of impurities such as lipids and proteins in the matrix, exacerbating the matrix effect in subsequent testing.

After optimization of the extraction solvent and time, it was found that acetonitrile–PBS (20:80, *v*/*v*) with 5 min extraction yielded the highest efficiency, ensuring maximal recovery of triazole pesticides for subsequent analysis.

### 3.4. Optimization of Reaction System

The concentration of antigen and antibody has a significant impact on the sensitivity of the chemiluminescence enzyme immunoassay. In order to improve detection sensitivity effectively while reducing detection costs, the chessboard method was employed to optimize the dilutions. Serial dilutions of antibody and biotinylated antigen solutions were prepared, and B/B_0_ ratios of signal in the presence versus absence of analyte were calculated for each pesticide. In general, the smaller the ratio, the higher the sensitivity. Considering the luminescence value and sensitivity, a dilution factor with appropriate luminescence value and sensitivity was selected.

As shown in Table 2, the optimal dilutions were 1:8000 for the triazolone monoclonal antibody solution and 1:20,000 for the biotinylated triazolone solution, and 1:4000 for the tebuconazole monoclonal antibody solution, with 1:20,000 for the biotinylated tebuconazole solution, respectively. At this dilution ratio, the reaction system may have higher sensitivity.

### 3.5. Method Performance Evaluation

#### 3.5.1. Establishment of Standard Curve

**Figure 7:**
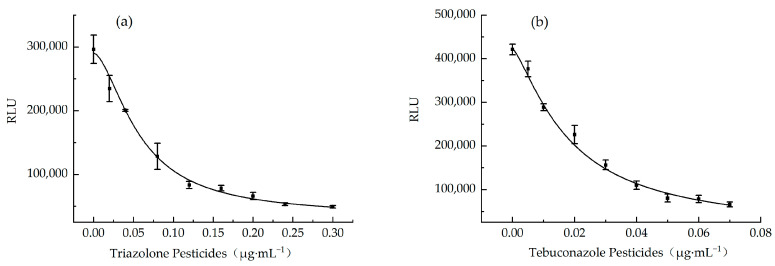
Standard curves of two pesticides in wheat. (**a**) Triazolone and (**b**) tebuconazole.

To establish standard curves, chemiluminescence values were measured for samples spiked at seven different concentrations. As shown in Figure 7, there was a good dose–response relationship between the concentration of pesticides and the corresponding RLU. The the standard curve of triazolone was Y_triazolone_ = 34,344 + (290,234 − 34,344)/[1 + (x/0.05747)^1.70248^], the correlation coefficient R^2^ = 0.998. The standard curve of tebuconazole was Y_tebuconazole_ = 4554 + (422,412 − 4554)/[1 + (x/0.01841)^1.3398^], and the correlation coefficient R^2^ = 0.994. The R^2^ of the standard curve of the two kinds of pesticides was higher than 0.99, indicating that the standard curve was accurate and reliable.

#### 3.5.2. Sensitivity Evaluation

After condition optimization, the method was further developed. As shown in Table 3, under optimized conditions, LODs were determined to be 0.002835 μg·mL^−1^ for triazolone and 0.00064 μg·mL^−1^ for tebuconazole in wheat, while LOQs were far below the national standard. It indicated that the method had high sensitivity and could detect trace triazole residue in wheat samples.

#### 3.5.3. Precision Evaluation

To evaluate method precision, blank wheat samples (5.0 ± 0.05 g) were spiked with triazolone at 0.05, 0.1, and 0.2 μg·mL^−1^ and tebuconazole at 0.0125, 0.025, and 0.05 μg·mL^−1^, then extracted and analyzed as described above. The results showed that the RSD (*n* = 7) were below 10% in Table 4, indicating that the established method for both pesticides had good precision and the results of repeated detection were stable, which could meet the requirements of actual detection.

#### 3.5.4. Accuracy Evaluation

**Figure 8:**
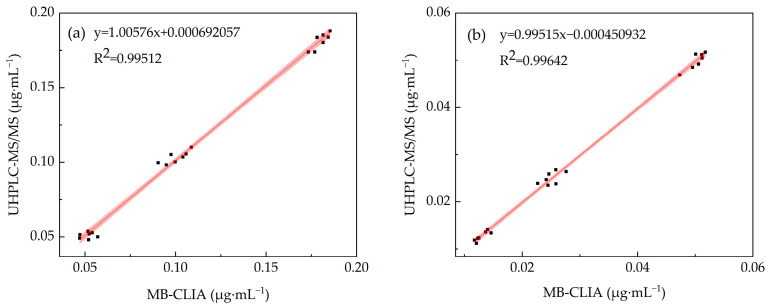
Comparison between MB-CLIA and UHPLC-MS/MS. (**a**) Triazolone and (**b**) tebuconazole.

To verify method accuracy, the supernatant of blank wheat samples was spiked with triazolone and tebuconazole at 0.5×, 1×, and 2× the international maximum residue limits and then analyzed by MB-CLIA; the results were obtained by UHPLC-MS/MS [42]. As shown in Table 5, the recovery rates of MB-CLIA and UHPLC-MS/MS ranged from 90.1% to 103.6% and 90.7% to 103.3%, respectively, with coefficients of variance (CV) ranging from 2.4% to 8.5% and 3.1% to 8.1%. The above recovery rate and CV are within an acceptable range, indicating that the established method meets the requirements for quantitative analysis of triazolone and tebuconazole. As shown in Figure 8, by establishing a linear equation (Y represents the concentration determined by the UHPLC-MS/MS method, and X represents the concentration determined by the MB-CLIA method), the correlation coefficient was calculated to be greater than 0.99. Figure 8a shows that the 95% confidence intervals (CI) for the slope (b = 1.00576, 95% CI: 0.9728–1.03872) and intercept (a = 0.000692057, 95% CI: −0.00335–0.00474) include ideal values of 1 and 0, respectively. Figure 8b shows that the 95% confidence intervals (CI) for the slope (b = 0.99515, 95% CI: 0.96722–1.02308) and intercept (a = 0.0000450932, 95% CI: −0.000974054–0.000883868) also include ideal values of 1 and 0, respectively, indicating that there is no proportional or systematic bias between the two methods. The detection results of the two methods showed a good correlation, indicating that the developed MB-CLIA is highly consistent with the standard instrument method, demonstrating the accuracy and practicality of the detection.

#### 3.5.5. Selectivity Evaluation

To evaluate specificity, cross-reactivity was assessed using five structural analogs: chlorthal, dichlorvonitrile, difenoconazole, diniconazole, and pentachloroaniline. As shown in Table 6, cross-reactivity rates were less than 0.1% for both pesticides, indicating negligible interference and excellent specificity for detecting these residues in wheat.

#### 3.5.6. Wheat Sample Detection

In the actual sample detection, neither triazolone nor tebuconazole was detected by this method and UHPLC-MS/MS, and the results showed good consistency. This method has been proven to have high reliability and practicality.

## 4. Conclusions

In summary, MB has advantages such as a large specific surface area, supermagnetism, and excellent biocompatibility. It was used as a carrier for indirect competition in CLIA and manipulated through the use of external magnetic fields from integrated devices. Subsequently, an MB-based CLIA was developed and applied to the detection of triazole pesticide residues in wheat samples. Sample pretreatment is a primary factor for enriching target substances and minimizing matrix interference [43]. Through the optimization of pre-treatment conditions and the reaction system, the optimal detection conditions were established. The detection limits of triazolone and tebuconazole in wheat were 0.002835 μg·mL^−1^ and 0.00064 μg·mL^−1^, respectively, with recoveries ranging from 90.1% to 103.6%. The method was accurate and reliable. RSD < 10% showed good precision. The cross-reaction rate of the two pesticides with their structural analogs was less than 0.1%, showing good specificity. The method has the advantages of high accuracy, good repeatability, and high throughput, which could realize the rapid quantitative detection of triazole pesticides in wheat samples in a short time, providing a new solution for the detection of pesticide residues in grain, and has broad application prospects. In future food-quality and safety-inspection work, it is expected to play an important role in providing strong technical support for ensuring food security and human health.

## Figures and Tables

**Table 1:** Analytical parameters and retention time for the determination of triazolone and tebuconazole with the UHPLC-MS/MS.

Analyte	Retention Time/min	MRM Transition	Quantifier/Qualifier	CE/V	DP/V
Triazolone	7.01	294.1–225.1	Quantifier	15	80
		294.1–197.1	Qualifier	22	80
Tebuconazole	7.43	308.1–151.1	Quantifier	35	80
		310.1–127.1	Qualifier	55	80

**Table 2:** Effect of antigen–antibody dilution ratio on the triazolone sensitivity in wheat matrix (*n* = 3).

**(1) Effect of antigen–antibody dilution ratio on the triazolone sensitivity in wheat matrix (*n* = 3).**				
**Antigen Dilution**	**Antibody Dilution**			
	**1:2000**	**1:4000**	**1:6000**	**1:8000**
1:10,000	0.57	0.57	0.47	0.56
1:12,500	0.58	0.51	0.44	0.55
1:15,000	0.51	0.49	0.42	0.42
1:20,000	0.50	0.46	0.37	**0.36**
**(2) Effect of antigen–antibody dilution ratio on the tebuconazole sensitivity in wheat matrix (*n* = 3).**				
**Antigen Dilution**	**Antibody Dilution**			
	**1:1000**	**1:2000**	**1:4000**	**1:6000**
1:5000	0.72	0.66	0.58	0.56
1:10,000	0.68	0.60	0.54	0.53
1:15,000	0.58	0.53	0.45	0.47
1:20,000	0.54	0.49	**0.43**	0.45

**Table 3:** The detected limit of triazolone and tebuconazole in the wheat blank.

	Triazolone/ μg·mL^−1^	Tebuconazole/ μg·mL^−1^
Average	0.0008200	0.0002025
SD	0.0006725	0.0001450
LOD	0.0028350	0.0006400
LOQ	0.0075450	0.0016525

**Table 4:** Test precision of the method (*n* = 7).

Pesticides	Spiked/ μg·mL^−1^	RLU Value							RSD/%
		1	2	3	4	5	6	7	
Triazolone	0.0000	301,135	289,002	291,783	301,825	273,961	289,467	292,766	3.2
	0.0500	173,294	163,296	183,961	172,697	174,061	169,201	183,661	4.3
	0.1000	106,320	115,290	101,265	99,027	110,756	102,656	108,346	5.4
	0.2000	66,892	68,247	67,248	66,024	65,288	65,024	66,024	1.7
Tebuconazole	0.0000	431,067	412,942	405,817	424,917	405,901	433,886	419,688	2.7
	0.0125	275,247	266,912	246,028	251,708	271,378	254,869	269,078	4.3
	0.0250	175,924	166,923	184,609	174,249	173,402	158,217	167,042	4.9
	0.0500	91,248	96,589	92,247	89,347	88,248	90,347	89,235	3.1

**Table 5:** Recovery test for MB-CLIA and UHPLC-MS/MS. (*n* = 7).

Pesticides	Spiked/ μg·mL^−1^	MB-CLIA			UHPLC-MS/MS		
		Measured/ μg·mL^−1^ (Mean ± SD)	Recovery/%	CV/%	Measured/ μg·mL^−1^ (Mean ± SD)	Recovery/%	CV/%
Triazolone	0.0500	0.0515 ± 0.0034	103.1	6.5	0.0511 ± 0.0020	102.3	3.9
	0.1000	0.1006 ± 0.0065	100.1	6.5	0.1033 ± 0.0041	103.3	4.0
	0.2000	0.1801 ± 0.0043	90.1	2.4	0.1813 ± 0.0056	90.7	3.1
Tebuconazole	0.0125	0.0129 ± 0.0011	103.6	8.5	0.0127 ± 0.0010	101.6	8.1
	0.0250	0.0250 ± 0.0015	100.1	6.1	0.0250 ± 0.0014	100.0	5.4
	0.0500	0.0502 ± 0.0015	100.4	3.0	0.0499 ± 0.0018	99.8	3.5

**Table 6:** Cross-reaction rates of triazolone and tebuconazole corresponding to structural analogs, respectively.

Pesticides	IC_50_ /μg·mL^−1^	CR/%	Pesticides	IC_50_ /μg·mL^−1^	CR/%
triazolone	0.065	100	tebuconazole	0.019	100
chlorthal	>100	<0.07	chlorthal	>100	<0.02
dichlorvonitrile	>100	<0.07	dichlorvonitrile	>100	<0.02
difenoconazole	>100	<0.07	difenoconazole	>100	<0.02
diniconazole	>100	<0.07	diniconazole	>100	<0.02
pentachloroaniline	>100	<0.07	pentachloroaniline	>100	<0.02

## Data Availability

The original contributions presented in this study are included in the article. Further inquiries can be directed to the corresponding authors.

## Abbreviations

The following abbreviations are used in this manuscript:

AP	Alkaline phosphatase
MB-SA	Magnetic bead-labeled streptavidin
CLIA	Chemiluminescence immunoassay
